# Novel Portable Sensing System with Integrated Multifunctionality for Accurate Detection of Salivary Uric Acid

**DOI:** 10.3390/bios11070242

**Published:** 2021-07-16

**Authors:** Ziqi Liu, Yiyin Chen, Meng Zhang, Tiancheng Sun, Keer Li, Songjia Han, Hui-Jiuan Chen

**Affiliations:** School of Electronics and Information Technology, Sun Yat-sen University, Guangzhou 510006, China; liuzq28@mail2.sysu.edu.cn (Z.L.); hxkqchenyiyin@163.com (Y.C.); zhangm378@mail.sysu.edu.cn (M.Z.); suntch@mail2.sysu.edu.cn (T.S.); liker@mail2.sysu.edu.cn (K.L.); hsongjia@mail.sysu.edu.cn (S.H.)

**Keywords:** accurate detection of saliva, portable salivary sensing, suction filtration, uric acid sensor, temperature compensation

## Abstract

Uric acid, as the terminal product of purine metabolism in the body, is an important marker of many diseases. Uric acid is abundant in saliva, offering the possibility of its non-invasive detection. However, it is sensitive to interference in saliva by a variety of factors. A reliable method of processing saliva is centrifugation (CF), but the cost and size of equipment limit its use in everyday life. In this study, a novel portable salivary-sensing system (PSSS) with integrated suction filtration (SF) and temperature insulation was proposed to obtain more accurate salivary uric acid levels through a simple procedure. The PSSS includes a saliva container, a high-sensitive uric acid sensor (UAS), an accompanying printed circuit board (PCB), and a mobile application. The responses produced by the UAS presents excellent linearity (4.6 μA/mM with R^2^ = 0.9964), selectivity, reproducibility, and stability for the detection of low levels of uric acid. The difference in detection values between the UAS and the commercial sensor is only ~4%. The primary feature of the saliva container is the processing of saliva by SF instead of CF. Samples from CF and SF showed no significant differences regarding uric acid levels, and both exhibited approximately 50% deviation from the untreated samples, while the difference in uric acid levels between the samples after SF and after applying both treatments was ~10%. Besides, insulation of the saliva container can partially eliminate sources of error induced by the environment during uric acid level testing. The PSSS provides a novel strategy for the immediate detection of specific markers in saliva. We believe that the PSSS has promising potential for future application in the rapid saliva testing.

## 1. Introduction

With the rapid evolution in sensing technology, analysis of human exocrine fluids is being considered as a promising non-invasive alternative to blood analysis [1]. Saliva is a complex mixture of fluids whose main components include proteins, urea, uric acid, microorganisms, enzymes, and a multitude of electrolytes [2]. Saliva is one of the most readily accessible human exocrine fluids, a healthy human body secretes 1–1.5 L of saliva per day through salivary glands [3]. It has been well demonstrated that the levels of certain markers in saliva correlate significantly with the levels of their counterparts in the blood [4,5,6,7,8]. Hence, saliva can be considered as “extracorporeal blood” that can be analyzed to reflect the physiological status of the body without invasive approach [9,10,11].

Uric acid is the most abundant antioxidant compound in saliva, as well as the terminal product of purine metabolism in the body [12]. Numerous studies in recent years have shown that salivary uric acid levels can be a diagnostic indicator for a wide range of diseases, including gout [13], cancer [14], diabetes [15], Lesch–Nyhan syndrome [16], hypertension [17], metabolic syndrome [18], and oral lichen planus [19]. In view of this, several portable sensors have been developed for the immediate detection of uric acid level in saliva. Kim et al. proposed an integrated wireless mouthguard biosensor to achieve real-time monitoring of uric acid levels in saliva [20]. Other studies focused on improving the sensitivity of uric acid sensors [21], or reducing costs [22]. However, almost all of these efforts were solely dedicated to improving the performance of the sensors themselves. The abundance of proteins, bacteria, cells, and low uric acid levels (typically those not exceeding 1 mM in patients with hyperuricemia [20]) in saliva may lead to inaccurate test results and ultimately misdiagnosis [1], which have been largely ignored. Potential gingival bleeding, as well as food and drink residues within the oral cavity may also contribute to false results. Furthermore, almost all salivary uric acid sensors so far are based on uricase, whose activity is very sensitive to temperature. Although the human oral cavity is usually at a constant temperature, in certain cold environments, the temperature of the harvested saliva drops dramatically in a short time, most likely resulting in pseudo low uric acid test levels [23]. To overcome these difficulties, a way to improve the accuracy of the measurement by removing cells, bacteria, and macromolecules using centrifugation (CF), inspired by the idea of blood sample processing, is worth considering [24]. However, a prerequisite for implementing this approach is centrifugal equipment, which undoubtedly annihilates the portability of the uric-acid-sensing system. Besides, the usually high cost of centrifugal equipment prohibits its application in everyday life. Processing of saliva by a vacuum filtration apparatus is another mechanism to be considered. Common vacuum filtration apparatus creates a vacuum space by means of small manual pumps [25], but it is vulnerable to extreme temperatures. Therefore, there is an urgent need for a low-cost and simple solution for purifying saliva samples under complex environments combined with a highly sensitive sensor for rapid detection.

**Figure 1:**
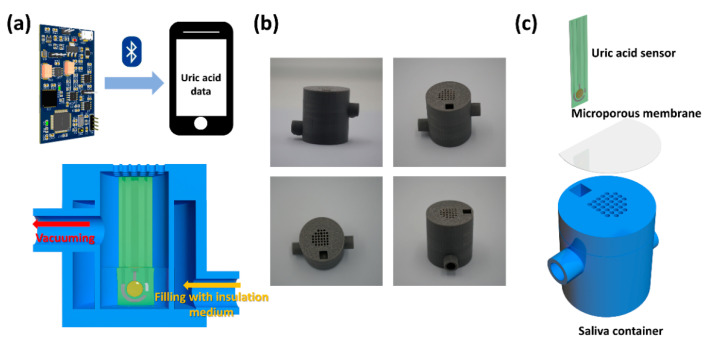
(**a**) Schematic diagram showing the structure and the application scenarios of the PSSS. (**b**) Photographs of saliva collection containers from different angles. (**c**) Assembly of the saliva container, microporous membrane, and the UAS.

With the aim of obtaining more accurate salivary uric acid levels conveniently, a portable salivary sensing system (PSSS) with integrated suction filtration (SF) and thermal insulation (TI) is presented in this study. The PSSS includes a 3D-printed saliva container, a high-sensitive uric acid sensor (UAS), a printed circuit board (PCB), and a mobile application. The container allows SF of saliva at a constant temperature and the UAS detects the purified saliva sample and sends the data to a smartphone via Bluetooth on the PCB. The application process of the whole system is shown in Figure 1a. The acquisition, processing, and detection process could all be completed in just a few minutes. Comparative experiments indicated that the purified saliva sample demonstrated significant differences in uric acid levels compared to the unprocessed sample. The PSSS is an alternative to CF and able to compensate for certain test biases induced by extreme low temperatures. The performance of the UAS in PSSS is essentially identical to that of commercial sensor (CS). For the detection of other biochemical indicators in saliva, the PSSS also exhibited the versatility to be a generic platform. Besides, the PSSS does not require contact with the oral cavity, thus ensuring the safety in its application. Because of its portability, low cost, and high accuracy, the PSSS has a promising potential to be applied in salivary analysis and disease diagnosis.

## 2. Materials and Methods

### 2.1. Samples and Reagents

All of the following reagents were acquired from Sigma-Aldrich (St Louis, USA): Uricase (4 U/mg, from *Candida* sp.), bovine serum albumin (BSA), glutaraldehyde solution (20–25%), uric acid (UA), glucose, L-ascorbic acid (AA), sodium chloride (NaCl), potassium chloride (KCl), and phosphate buffer saline (PBS, pH = 7.2–7.4). Artificial saliva (pH = 6.6–7.1) was obtained from Shanghai Yuanye Bio-Technology Co., Ltd. (Shanghai, China). All reagents were used without any modification. The microporous membrane (pore diameter = 220 nm) was purchased from Tianjin Jinteng Experimental Equipment Co., Ltd. (Tianjin, China). and used as received. The pore size distribution of the microporous membrane was measured by ImageJ (200 micropores were randomly selected from the SEM photographs).

### 2.2. Production of the Uric Acid Sensor

The uric acid sensor was produced based on a commercial three-electrode substrate (Ningbo Mxense Bio-Tech Co., Ltd., Ningbo, China) with a reference electrode of Ag/AgCl. The working and counter electrodes are made of carbon. As illustrated in Figure S1, first, 20 nm Cr/60 nm Au was deposited on the working electrode via magnetron sputtering to enhance the electrical conductivity. Following this, 4 μL of a solution consisting of uricase (30 mg/mL), BSA (30 mg/mL) and glutaraldehyde (15 mg/mL) was drop-casted on the working electrode area. The uric acid sensor was left to air dry at room temperature overnight.

### 2.3. Design, Fabrication, and Demonstration of Hardware and Software

The PCB relies heavily on an STM32F103RCT6 microcontroller and its attached circuits to perform all functions, and the schematic diagrams of all circuits are shown in Figure S2. In summary, the current signal generated by the uric acid sensor is processed by the uric acid signal acquisition circuit, and then sent by the Analog to Digital Converter (ADC) of the microcontroller to Bluetooth and finally transmitted to the mobile application. In order to increase the accuracy and sensitivity of uric acid detections, a digital-to-analog converter (DAC) of the microcontroller was employed to output a constant potential (0.6 V) to ensure the reduction of the hydrogen peroxide produced by the uric acid reaction. The sampling rate is set to 10 Hz by programming the microcontroller. The uric acid signal acquisition circuit can measure the current signal with an accuracy of up to 1 nA. For reducing energy consumption and prolonging the usage time of the system, low-power Bluetooth (RF-BM-4044B4, Shenzhen RF-star Technology Co., Ltd., Shenzhen, China) was adopted to achieve wireless data transmission. This low-power Bluetooth is small and provides stable and reliable data transmission within a few meters. A lithium battery with an output voltage of 3.7 V (Figure S3) was utilized to provide power to the PCB. With the PCB, the 3.7 V power supply can be properly converted to 5 V, −5 V, and 3.3 V to satisfy the power needs of individual modules.

The PCB is shown in Figure S4a after punching and placement were completed. The acquisition test of the simulated sensor current signal was carried out to examine whether the board meets the requirements of system application. As shown in Figure S4b, the output results of the PCB showed a good linear response to the current signal. As a terminal for data transmission, the data for the mobile application is obtained through JAVA programming, then displayed to the user interface as shown in Figure S5. The main interface not only displays the uric acid test value and the current Bluetooth connection status, but also provides the function of saving data. The final uric acid level (averaged over 10 s) is displayed on the interface 150 s after the start of the test.

### 2.4. Fabrication, Assembly, and Operation of the Portable Salivary Uric Acid-Sensing System

The saliva collection container was designed in Solidworks software and manufactured by a 3D printer (Formlabs Form3, Formlabs Inc., Somerville, USA). The uric acid sensor was connected to the PCB through a dedicated interface and subsequently inserted into the pre-set hole above the saliva collection container. Prior to operation, the side vessel of the saliva collection container was filled with 37 °C water through the inlet hole and its pumping hole was connected to a small manual vacuum pump. The microporous membrane was then placed over the porous vent at the top of the saliva collection container. As drops of saliva were added to the microporous membrane, the vacuum pump began to run, allowing the microporous membrane to attach completely to the saliva collection container. When the saliva was being filtrated, the mobile application was connected to the PCB via Bluetooth, and the uric acid sensor started testing for 150 s, the final data acquired was the average of 100 data points over 10 s.

### 2.5. Evaluation of In Vitro Performance of the Uric Acid Sensor

In vitro tests were performed in the PBS solution and an electrochemical workstation (CHI700E, CH Instruments Ins, Austin, USA) was employed. The linearity and responsiveness of the uric acid sensor were evaluated at uric acid concentration gradients of 0, 100, 200, 300, 400, and 500 μM. The reproducibility was evaluated by the distribution of the response values of five different uric acid sensors prepared under the same process for 100, 200, and 300 μM uric acid concentration gradients. The response values for a same uric acid sensor at 100, 300, and 500 μM uric acid concentration gradients were tested at Day 1, 2, 3, 4, and 10 to measure its temporal stability. The selectivity of the uric acid sensor was assessed by testing the response values of sequentially adding 400 μM UA, 500 μM glucose, 5 mM LA, 50 μM AA, 10 mM KCl, and 10 mM NaCl to PBS.

### 2.6. Response Value Correction Experiment under Low-Temperature Environment

To help validate the calibration of the portable salivary uric-acid-sensing system at low temperatures, a cold test environment was simulated. Artificial saliva was added to the portable salivary uric-acid-sensing system filled with air and 37 °C water in the side vessel. The systems were then maintained at 4 °C environment for 15 min. About 0, 10, 20, 30, and 40 μM UA were added sequentially to the artificial saliva in the system, for which the response values were tested.

### 2.7. Evaluation of Practical Application Potential of the Portable Salivary Uric Acid Sensor System

To evaluate the variability between different treatments and the detection performance of the uric acid sensor in practical applications, human saliva samples were harvested and tested after different treatments. Human saliva samples were collected using the “passive drool” method [26], with consents given by the participants allowing all samples collected. To explore the dissimilarity between centrifugation and suction filtration, saliva samples were collected from six subjects (S1–S6). Each sample was divided into three groups and processed by no-treatment, centrifugation, or suction filtration through the portable salivary uric-acid-sensor system, followed by detection by the uric acid sensor. To further assess the substitutability of the portable salivary uric-acid-sensor system for centrifugation, saliva samples were collected from three other subjects (S7–S9), and each sample was divided into three groups and processed by either centrifugation, or suction filtration through the portable salivary uric-acid-sensor system, or both treatments collectively, followed by the uric acid sensor. Each of the above samples was tested three times and the average value was calculated to ensure accuracy. The no-treatment saliva of all nine samples was tested with uric acid sensor and commercial uric acid sensor (EA-11, Sinocare Inc., Changsha, China) to verify the detection performance of uric acid sensor in saliva samples. The difference between these treatments and detection methods and the consistency were tested by least-significant difference (LSD) and intraclass correlation coefficient (ICC) via SPSS software.

## 3. Results and Discussion

### 3.1. Structure and Rationale of the PSSS

The detection of uric acid has evolved into several forms of signal transduction [21,27,28]. Bearing the advantages of compact size, competitive cost, and high sensitivity, electrochemical method is currently the most popular way to detect uric acid [29]. To avoid interference of electrochemical signals by electroactive substances with similar oxidative potentials in biological fluids, the sensitivity of uricase is extremely high [30]. The essential mechanism of uricase detection relies on the oxidation of uric acid to allatonin and hydrogen peroxide, which allows it to generate a current signal through a reduction reaction. Although there have been reports of the utilization of Prussian blue as a catalytic medium to reduce the potential required for hydrogen peroxide reduction, the relative complexity of the preparation process diminishes the reliability of its preparation [31]. Therefore, the detection method of reducing hydrogen peroxide at high potentials (0.6 V) was selected. Despite this, there are still challenges to overcome when using the electrochemical detection methods based on uric acid oxidase. The high viscosity of saliva and the abundance of macromolecules are not favorable to mass transfer and electrode stabilization. Therefore, there is an urgent need to improve the credibility by obtaining purified samples. Another challenge of using uric acid oxidase to detect uric acid is the fluctuation in the activity of uricase due to temperature changes, thus it is necessary to maintain a relatively stable temperature during testing in this newly developed integrated system, the ingenious sandwich structure allows the side vessel to be filled with an aqueous medium to maintain a certain temperature while retaining the convenience of evacuation. The smaller diameter of the internal container used to collect post-extraction saliva facilitates the detection especially for small volumes of samples. The photographs of the saliva container are shown in Figure 1b, and the schematic diagram of its assembly with the microporous membrane and the UAS is illustrated in Figure 1c.

**Figure 2:**
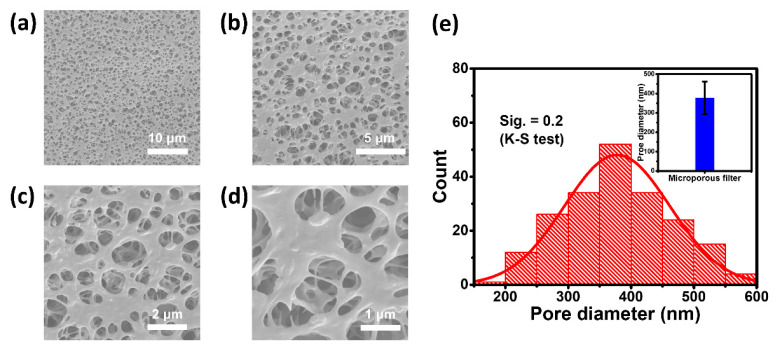
Characterization of the microporous membrane. (**a**–**d**) Typical SEM images of the microporous membrane at different magnifications. (**e**) Statistical results of the pore size distribution of the microporous membrane and its Kolmogorov–Smirnov test results (the inset shows the mean and standard deviation of pore size of the microporous membrane).

The effectiveness of SF is highly dependent on the pore size distribution of the commercial microporous membrane, which does not always meet the requirements precisely. Therefore, re-inspection and re-evaluation are deemed necessary. Figure 2a–d shows the scanning electron microscopy (SEM) results of the microporous membrane, characterized by numerous multilayer porous structures. About 200 micropores were randomly selected in SEM images for calculating statistics on pore sizes, and the results are shown in Figure 2e. The average pore size of the microporous membrane is approximately 378 nm with a standard deviation of 84 nm. The Kolmogorov–Smirnov test was adopted to assess the pore size distribution, with progressive significance (Sig.) = 0.2, indicating compliance with a normal distribution. A crucial fact is that the vast majority of bacteria are between 0.5 and 7 μm in size, with cell sizes being much larger, implying that the microporous membrane has the ability to remove bacteria and cells. The microscopic morphological characterization of the microporous membrane verified that the macroscopic and microscopic pore size distribution fulfill the requirements.

### 3.2. In Vitro Testing of the UAS

**Figure 3:**
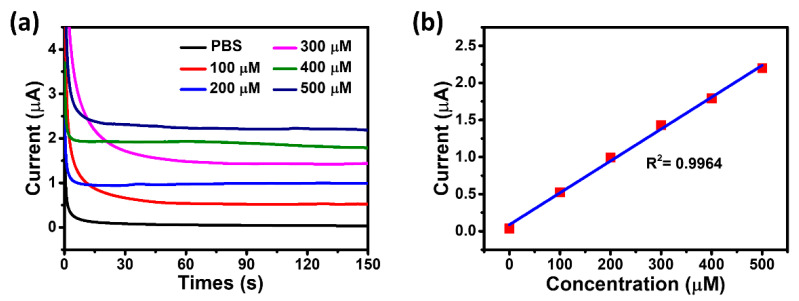
(**a**) The chronoamperometric responses of the UAS for increasing uric acid concentration with 100 μM increments up to 500 μM. (**b**) The linear calibration curve of the UAS.

Salivary uric acid levels in healthy individuals range from 100–250 μM [32]. Therefore, to summarize the changes in salivary uric acid levels, the electrochemical performance of the UAS was evaluated in a 100 μM concentration gradient of PBS solution (0–500 μM). As shown in Figure 3a, each test time was 150 s, and the average value over a duration of 10 s was taken to produce a linear calibration graph. Figure 3b demonstrated that the UAS has a very sensitive linear response to uric acid with a slope (sensitivity) of 4.6 μA/mM for the linear calibration plot and a correlation coefficient (R^2^) of 0.9964.

**Figure 4:**
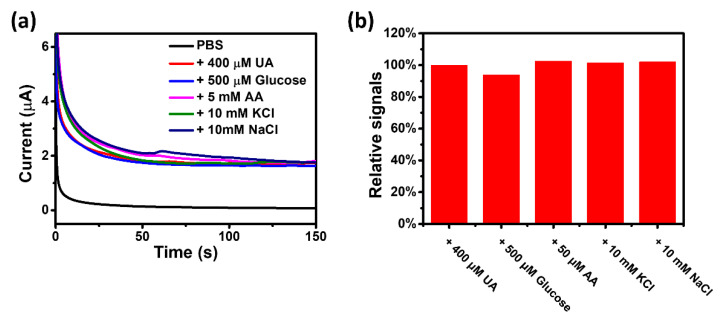
(**a**) The chronoamperometric responses of the UAS for adding uric acid and different interfering substances. (**b**) Statistical results of the effect of each interfering substance on the signal of the UAS.

Saliva is a complex fluid, containing a very large number of small interfering molecules even after CF or SF. Therefore, it is of great significance to assess the selectivity of the UAS. Common interfering substances in saliva, including glucose, ascorbic acid, potassium, and sodium, were selected to evaluate the selectivity of the UAS. Figure 4a illustrated that the UAS provided excellent responsiveness to 400 μM uric acid and negligible response signal to other interfering substances. Figure 4b quantitatively illustrated the effect of adding each interfering substance, while the relative signal values of the UASs were 93.9% for glucose, 102.5% for AA, 101.4% for KCl, and 102.1% for NaCl.

**Figure 5:**
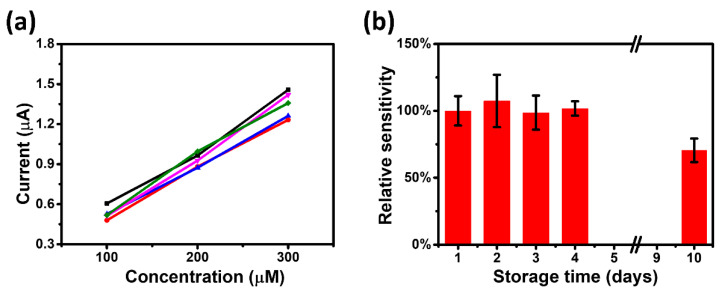
(**a**) The reproducibility of the UAS (five samples). (**b**) The time-stability of the UAS (the error bars were derived from the standard deviation of the measured data for five samples).

For mass-produced sensors, calibrating each UAS individually increases the complexity of use. Therefore, the reproducibility of the UAS needs to be ensured to eliminate the calibration process. Figure 5a displayed the reproducible experimental results of five UASs prepared under the same process, illustrating that their absolute response values are generally consistent. Furthermore, a single UAS is required to maintain acceptable stability over a certain period of time to fulfill the practical demands. A UAS was stored at room temperature and analyzed for response performance on Day 1, 2, 3, 4, and 10. As shown in Figure 5b, the sensitivity of the UAS remained essentially stable for 4 days and more than 70% of the original sensitivity was still maintained on Day 10. These results strongly suggest that the UAS has excellent reproducibility and stability over time.

### 3.3. Measurement Stability Evaluation in Low Temperature Environment

**Figure 6:**
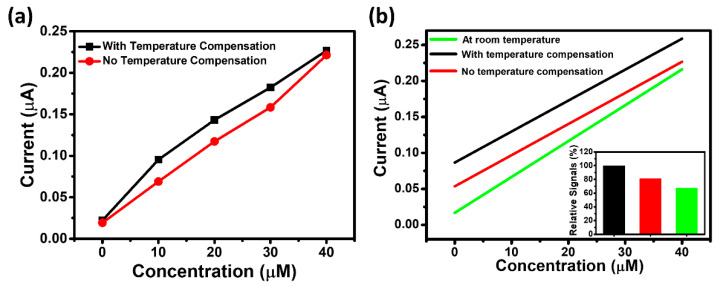
(**a**) Comparative results of uric acid test levels in cold environments with and without insulation from the PSSS. (**b**) Comparative fitting results of uric acid test levels in cold environments with and without the PSSS insulated, as well as at in room temperature (The inset shows the relative average signals for three conditions).

As described in the previous sections, the UAS may be used under a variety of environments, especially in cold regions where the accuracy of the UAS can be dramatically compromised. To evaluate the performance of the PSSS against low temperatures, comparative experiments in cold environments were conducted. Artificial saliva with different uric acid concentrations were employed to simulate saliva from patients. A higher water temperature may destroy the enzyme activity, while a lower water temperature could reduce the thermal insulation performance. Considering that the temperature of fresh saliva is ~37 °C, we chose 37 °C water as the insulation layer to maintain the current temperature of saliva as much as possible. In addition, the uric acid concentrations were set very low to assess the accuracy of the PSSS under slight changes in uric acid levels. The comparative results of testing through the PSSS with and without thermal insulation are shown in Figure 6a. The response signals with thermal insulation were higher, indicating that the thermal insulation process increased the activity of uricase. The linear fit results were compared between the insulated, uninsulated, and room temperature. As illustrated in Figure 6b. The response signals from the PSSS are much closer to those tested at room temperature than the uninsulated one. The difference in the slope of the response signals at room temperature and after insulation compensation is mainly at the intercepts, which may be caused by small differences in different UASs. These results further demonstrate the ability of the PSSS to compensate for errors caused by temperature to some extent.

### 3.4. Evaluation of the PSSS for Practical Applications

**Figure 7:**
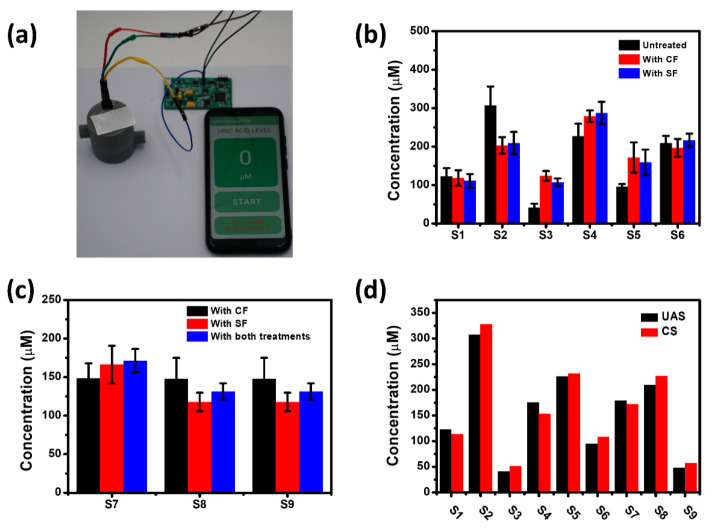
(**a**) Photograph of the PSSS application scenario. (**b**) Uric acid levels in six groups (S1–S6) of real saliva samples after unprocessed, CF, and SF through the PSSS, respectively. (**c**) Uric acid levels in three groups (S7–S9) of real saliva samples after CF, SF, and both treatments through the PSSS, respectively (The error bars in (**b**,**c**) are obtained from the standard deviation of the data measured three times per sample). (**d**) Uric acid levels in all nine groups (S1–S9) of real saliva samples tested by the UAS of the PSSS and CS, respectively.

One of the most important objectives of designing the PSSS is to carry out the CF under simple conditions in order to obtain results that more closely approximate real uric acid levels, and a variety of approaches were utilized to evaluate the achievement of the objective. Nine groups of saliva samples from humans were collected, processed, and tested in different ways. All samples were harvested and held in saliva container. The scenarios for the utilization of the PSSS are shown in Figure 7a, and the footprint of the entire system (excluding the manual vacuum pump) is no more than the size of a cell phone. Figure 7b displayed the uric acid levels for each of the six groups (S1–S6) of real saliva samples that were processed by no-treatment, CF, and SF through the PSSS, respectively. Owing to the variability of saliva samples from different individuals, salivary uric acid levels may not always change after CF or SF. The uric acid levels of most samples were significantly different between no-treatment and after CF or SF, while the centrifuged and filtrated samples were mostly similar. Figure 7c compared the uric acid levels of saliva samples (S1–S7) after CF, SF, and both treatments to assess the similarity of CF and SF from another perspective. The saliva samples after SF and CF still demonstrated relatively close uric acid levels, with also no significant changes in the samples after applying both treatments. To demonstrate the reliability of the PSSS for the above test results, untreated saliva samples from S1–S9 were also tested for comparison using the UAS of the PSSS and the CS, respectively, as shown in Figure 7d. According to the results, the test results of both sensors are relatively close, which tentatively demonstrate the adequate substitution of SF for CF.

**Figure 8:**
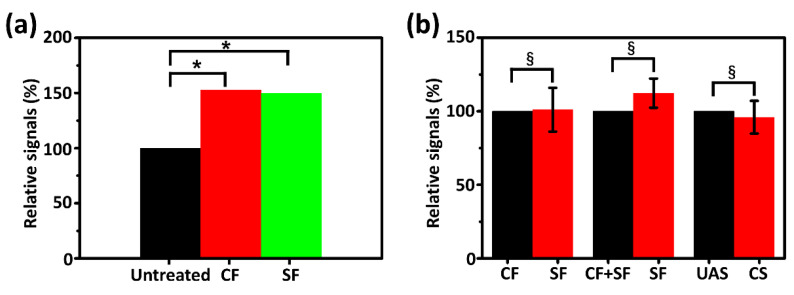
(**a**) Comparison of relative mean uric acid levels in real saliva samples after untreated, CF and SF. (**b**) Comparison of relative mean uric acid levels in real saliva samples with different treatments and assays: treated with CF and SF (left), treated with SF, and applying both the treatments (middle), and tested by the UAS and CS (right) (the error bars were obtained from the standard deviation of all previous measurements in the same category). * *p* < 0.05, ^§^ > 0.95.

For further justification of the above results, strong statistical evidence must be provided. First, the results in Figure 7b were averaged and the uric acid level of the untreated saliva sample was set to 100 to compare the relative uric acid levels of the other two groups, as shown in Figure 8a. Samples from CF and SF showed no significant differences regarding uric acid levels, and both of them exhibited approximately 50% deviation from the untreated samples. LSD was applied to assess the significant differences of the three samples from each other and the results are shown in Figure 8a and Table 1. The LSD of uric acid levels between centrifuged or filtered samples and untreated samples was less than 0.05, indicating a significant difference. The LSD of uric acid levels for the centrifuged and filtered treated samples was 0.895, indicating that there was no significant difference between the two groups, but this result does not yet indicate consistency.

To further illustrate the adequacy of the PSSS in processing saliva samples, the results of Figure 7c,d were averaged individually and the relative uric acid levels between the different groups of samples were compared (the uric acid level of groups being compared were set to 100), as shown in Figure 8b. As with the results in Figure 8a, there was little difference in uric acid levels between the centrifuged samples and filtered samples. The difference in uric acid levels between the samples after SF and after applying both the treatments was ~10%. The difference in detection values between the uric acid sensor in the PSSS and the CS is only ~4%. The ICC was employed to test the consistency between several groups of samples, as shown in Figure 8b and Table 2, the ICC for all three groups is very close to 1. The results of ICC indicate that SF and CF have the same treatment effect. Even when applying both the treatments, the uric acid levels in saliva samples were consistent with those after SF only. Thus, SF is capable to be a satisfactory substitute for CF for the detection of uric acid levels in saliva samples. It was demonstrated that the performance of the UAS remained highly consistent with that of CS. These results complement the previous results and provide strong evidence of the potential of the PSSS for practical applications. Furthermore, the evaluation results of the whole system may provide new insights into the coordination of salivary-sampling methods with purification methods [33].

## 4. Conclusions

This work describes a novel sensing system for rapid and accurate detection of actual uric acid levels in saliva under a complex environment. The main structure of the PSSS has several unique design purposes and is combined with small PCBs that transmit data to mobile phones for portable detection. The UAS, on the other hand, has demonstrated high selectivity, sensitivity, and stability, and is comparable to the performance of commercial sensors in practical assays. PSSS achieved the portability and low cost of the whole system through SF, which can overcome the disadvantages brought by CF. The PSSS takes into account the unique properties of saliva, making it the “key” to unlocking information about human health and has a promising outlook for everyday biomedical applications. Future refinements would mainly include further miniaturization of the circuit board and vacuum pump, as well as integration of the whole system. Moreover, the proposed system could be further modified for detecting other markers. Potential markers include glucose, lactate, etc., as they are present in saliva like uric acid and can be stably detected by electrochemical sensors [34].

## Figures and Tables

**Table 1:** Comparison of relative mean uric acid levels and LSD results in real saliva samples after untreated, CF and SF.

Groups	Relative Signals	LSD
CF/Untreated	152.84%	0.030 *
SF/Untreated	149.69%	0.041 *
SF/CF	97.04%	0.895

* *p* < 0.05.

**Table 2:** Comparison of relative mean uric acid levels and ICC results in real saliva samples with different treatments and assays: treated with CF and SF, treated with SF, and applying both the treatments, and tested by the UAS and CS.

Groups	Relative Signals	ICC
SF/CF	101.07%	0.984 ^§^
SF/(CF + SF)	112.33%	0.966 ^§^
UAS/CS	95.96%	0.994 ^§^

^§^ ICC > 0.95.

## Data Availability

Raw data presented in this study are available on request from the corresponding author.

## Supplementary Materials

The following are available online at https://www.mdpi.com/article/10.3390/bios11070242/s1. Figure S1: (a) Schematic diagram of the production process of the UAS. (b) Detail photograph of the UAS. Figure S2: Schematic diagram of all circuits in the PSSS. Figure S3: Detail photograph of the lithium battery (3.7 V) employed for supplying power. Figure S4: (a) Detail photograph of the PCB. (b) Input-output signal response for uric-acid-sensing signal channel of the PCB. Figure S5: Display interface for the mobile application. (a) The main interface. (b) The menu of functions. (c) Bluetooth scanning and connecting. (d) The uric acid level was displayed 150 s after the test started.
