# Supplementary material for: Novel Portable Sensing System with Integrated Multifunctionality for Accurate Detection of Salivary Uric Acid
Source: Biosensors (Basel). 2021 Jul 16;11(7):242. doi: 10.3390/bios11070242 (PMC8301860; doi:10.3390/bios11070242)
Supplement: Supplementary file 1 [file biosensors-11-00242-s001.zip › biosensors-1282481-supplementary.pdf]

Novel Portable Sensing System with Integrated Multifunctionality for Accurate Detection of Salivary Uric Acid

Ziqi Liu [1,†], Yiyin Chen [2,†], Meng Zhang [3],Tiancheng Sun [1], Keer Li [1], Songjia Han [1] and Hui-Jiuan Chen [1,*]

[1] State Key Laboratory of Optoelectronic Materials and Technologies, School of Electronics and Information Technology, Sun Yat-sen University, Guangzhou 510006, China; liuzq28@mail2.sysu.edu.cn (Z.L.); suntch@mail2.sysu.edu.cn (T.S.); liker@mail2.sysu.edu.cn (K.L.); hansongjia@126.com (S.H.)
[2] West China School of Stomatology, Sichuan University, Chengdu 610041, China; hxkqchenyiyin@163.com
[3] Precision Medicine Institute, The First Affiliated Hospital of Sun Yat-Sen University, Sun Yat-Sen University, Guangzhou 510080, China; meng.zhang_china@outlook.com
* Correspondence: chenhuix5@mail.sysu.edu.cn
† The two authors contributed equally to this paper

Supplementary

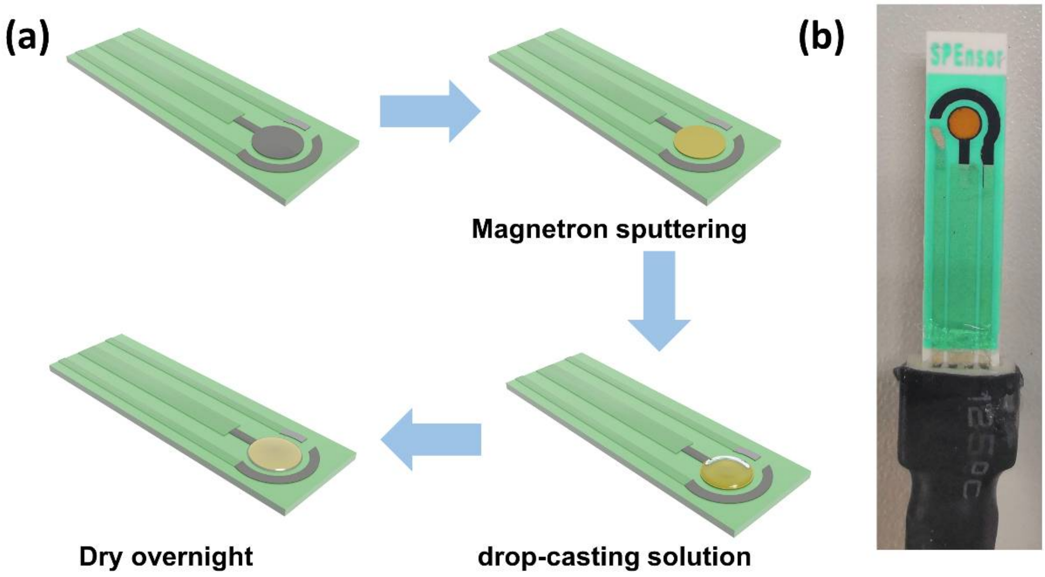

Figure S1. (**a**) Schematic diagram of the production process of the UAS. (**b**) Detail photograph of the UAS.

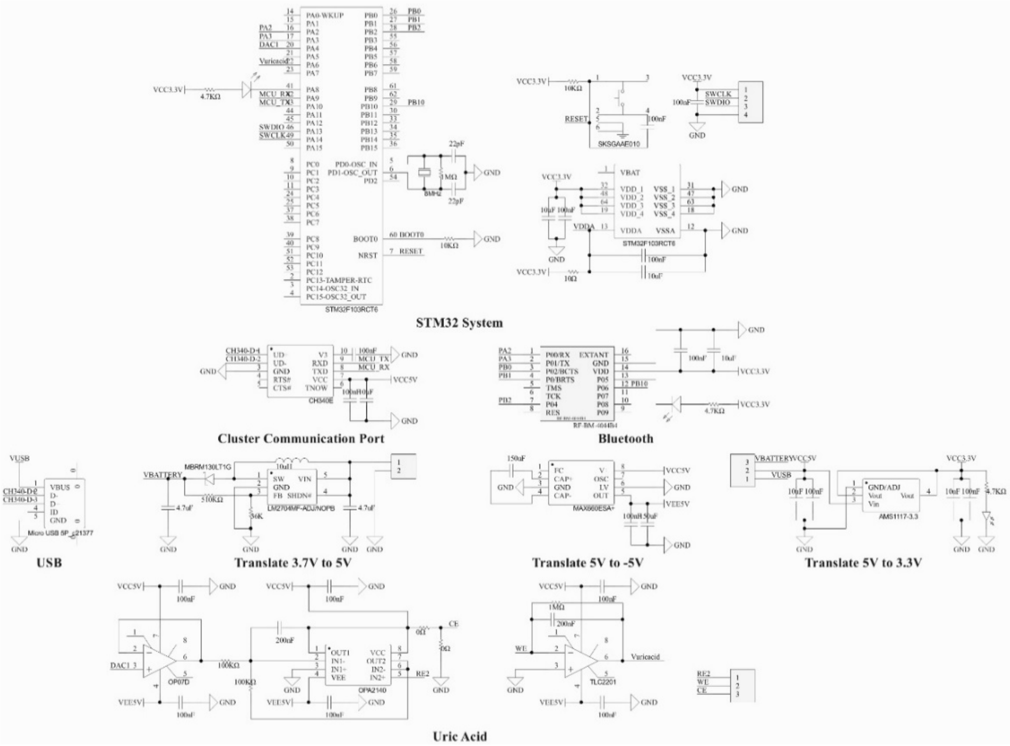

Figure S2. Schematic diagram of all circuits in the PSSS.

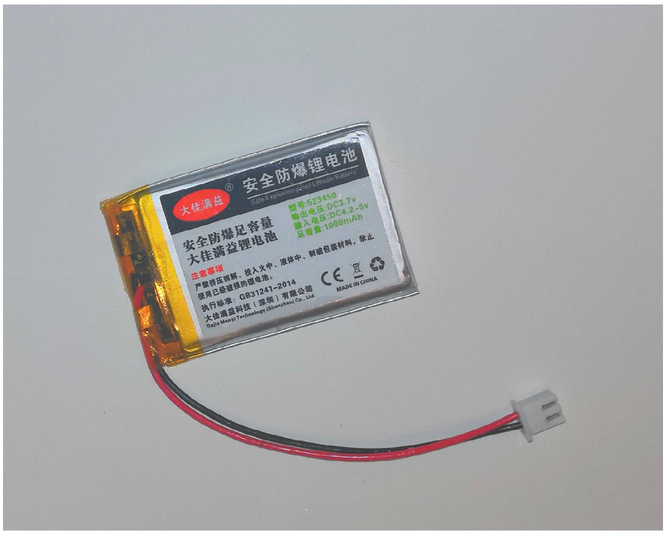

Figure S3. Detail photograph of the lithium battery (3.7V) employed for supplying power.

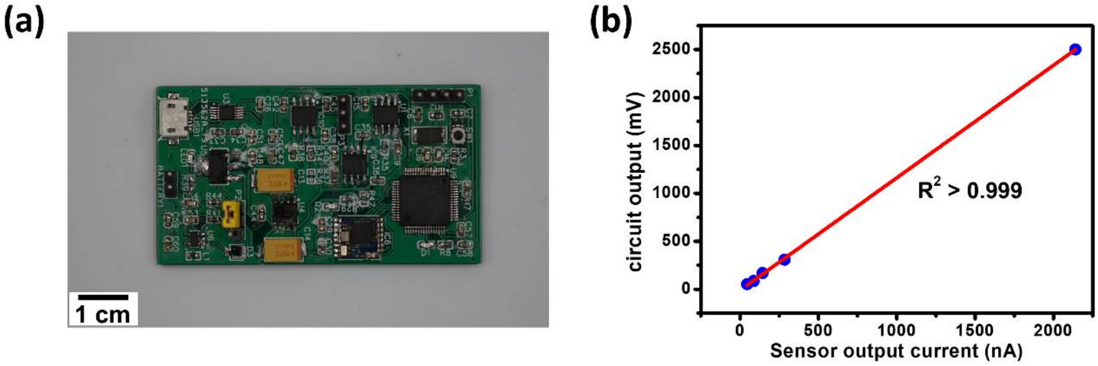

Figure S4. (**a**) Detail photograph of the PCB. (**b**) Input-output signal response for uric acid sensing signal channel of the PCB.

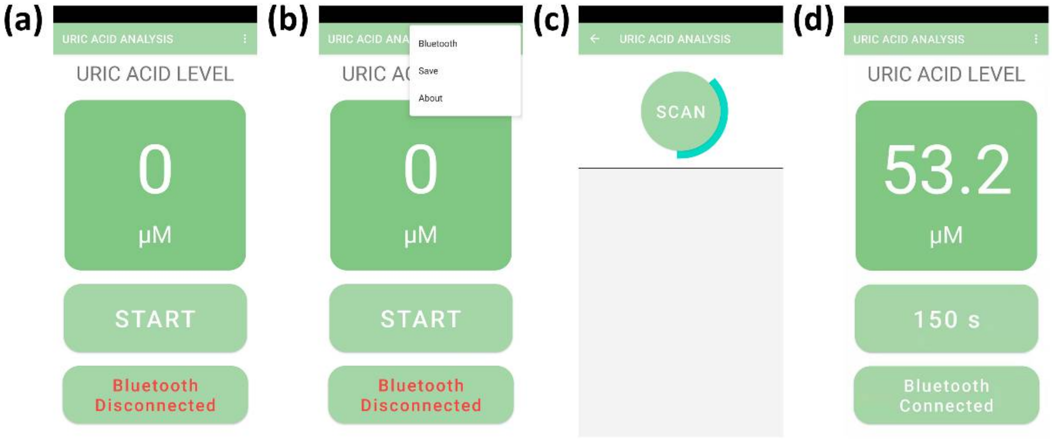

Figure S5. Display interface for the mobile application. (**a**) The main interface. (**b**) The menu of functions. (**c**) Bluetooth scanning and connecting. (**d**) The uric acid level was displayed 150s after the test started.
